# Thermostable D-amino acid decarboxylases derived from *Thermotoga maritima* diaminopimelate decarboxylase

**DOI:** 10.1093/protein/gzab016

**Published:** 2021-07-13

**Authors:** Antonija Marjanovic, Carlos J Ramírez-Palacios, Marcelo F Masman, Jeroen Drenth, Marleen Otzen, Siewert-Jan Marrink, Dick B Janssen

**Affiliations:** Biotechnology and Biocatalysis, Groningen Biomolecular Sciences and Biotechnology Institute, University of Groningen, Nijenborgh 4, 9747 AG Groningen, The Netherlands; Biotechnology and Biocatalysis, Groningen Biomolecular Sciences and Biotechnology Institute, University of Groningen, Nijenborgh 4, 9747 AG Groningen, The Netherlands; Molecular Dynamics Group, Groningen Biomolecular Sciences and Biotechnology Institute, University of Groningen, Nijenborgh 7, 9747 AG Groningen, The Netherlands; Biotechnology and Biocatalysis, Groningen Biomolecular Sciences and Biotechnology Institute, University of Groningen, Nijenborgh 4, 9747 AG Groningen, The Netherlands; Molecular Dynamics Group, Groningen Biomolecular Sciences and Biotechnology Institute, University of Groningen, Nijenborgh 7, 9747 AG Groningen, The Netherlands; Van’t Hoff Institute for Molecular Sciences, HIMS-Biocat, University of Amsterdam, Science Park 904, 1098 XH Amsterdam, The Netherlands; Biotechnology and Biocatalysis, Groningen Biomolecular Sciences and Biotechnology Institute, University of Groningen, Nijenborgh 4, 9747 AG Groningen, The Netherlands; Biotechnology and Biocatalysis, Groningen Biomolecular Sciences and Biotechnology Institute, University of Groningen, Nijenborgh 4, 9747 AG Groningen, The Netherlands; Molecular Dynamics Group, Groningen Biomolecular Sciences and Biotechnology Institute, University of Groningen, Nijenborgh 7, 9747 AG Groningen, The Netherlands; Biotechnology and Biocatalysis, Groningen Biomolecular Sciences and Biotechnology Institute, University of Groningen, Nijenborgh 4, 9747 AG Groningen, The Netherlands

**Keywords:** 6-aminocaproic acid, computational redesign, D-amino acid, decarboxylase, molecular dynamics

## Abstract

Diaminopimelate decarboxylases (DAPDCs) are highly selective enzymes that catalyze the common final step in different lysine biosynthetic pathways, i.e. the conversion of *meso*-diaminopimelate (DAP) to L-lysine. We examined the modification of the substrate specificity of the thermostable decarboxylase from *Thermotoga maritima* with the aim to introduce activity with 2-aminopimelic acid (2-APA) since its decarboxylation leads to 6-aminocaproic acid (6-ACA), a building block for the synthesis of nylon-6. Structure-based mutagenesis of the distal carboxylate binding site resulted in a set of enzyme variants with new activities toward different D-amino acids. One of the mutants (E315T) had lost most of its activity toward DAP and primarily acted as a 2-APA decarboxylase. We next used computational modeling to explain the observed shift in catalytic activities of the mutants. The results suggest that predictive computational protocols can support the redesign of the catalytic properties of this class of decarboxylating PLP-dependent enzymes.

## Introduction

Amino acid decarboxylases play a crucial role in various catabolic and biosynthetic processes, such as putrefaction and production of hormones and neurotransmitters (Li *et al.*, 2012). Two main groups of decarboxylases act on α-amino acids, i.e. prokaryotic pyruvoyl-dependent decarboxylases and the more widespread pyridoxal 5′-phosphate (PLP)-dependent decarboxylases. Examples of pyruvoyl-dependent decarboxylases include histidine decarboxylases, which produce the neurotransmitter histamine, and aspartate decarboxylases, which produce β-alanine, a precursor of pantothenate and coenzyme A. PLP-dependent decarboxylases have been classified in four groups (Sandmeier *et al.*, 1994; Liang *et al.*, 2019) and are found in the fold-type I, fold-type II and fold-type IV PLP-dependent enzymes (Grishin *et al.*, 1995). Aromatic α-amino acid decarboxylases belong to fold-type I PLP enzymes. This group includes DOPA decarboxylases involved in dopamine synthesis (Giardina *et al.*, 2011). Glutamate decarboxylases, which produce γ-aminobutyric acid (GABA), also belong to the fold-type I enzymes (Fenalti *et al.*, 2007). Most of these α-amino acid decarboxylases act on an L-stereocenter, converting L-amino acids to amines (Paiardini *et al.*, 2017).

Unlike the group I α-amino acid decarboxylases, the diaminopimelate decarboxylases (DAPDCs) (EC number: 4.1.1.20) act on a D-stereocenter. More specifically, they decarboxylate *meso*-diaminopimelic acid (DAP), the last intermediate in the biosynthesis of L-lysine. DAP is also a component of the bacterial cell wall in Gram-negative bacteria. Bacterial DAPDCs belong to the fold-type III dimeric PLP-dependent enzymes (alanine racemase family) (Grishin *et al.*, 1995; Jansonius, 1998; Kern *et al.*, 1999; Schneider *et al.*, 2000; Peverelli *et al.*, 2016; Liang *et al.*, 2019). These decarboxylases are related to L-ornithine decarboxylases (ODC) and L-arginine decarboxylases (ADC), forming the group II decarboxylases (Kidron *et al.*, 2007; Paiardini *et al.*, 2017). Four different L-lysine synthesis routes have been identified in plants and bacteria, all four starting from L-aspartate (Fig. S1) (McCoy *et al.*, 2006; Triassi *et al.*, 2014). DAPDC catalyzes the common last step, which is decarboxylation of DAP at the D-stereocenter to yield L-lysine. These enzymes are highly regulated and have a narrow substrate range. Humans lack the capacity to synthesize L-lysine via any of the four pathways and are bound to obtain it from dietary sources. DAPDCs are therefore potential targets for development of drugs that act against pathogenic bacteria such as *Staphylococcus aureus* or *Mycobacterium tuberculosis* (Pavelka and Jacobs, 1996).

**Fig. 1:**
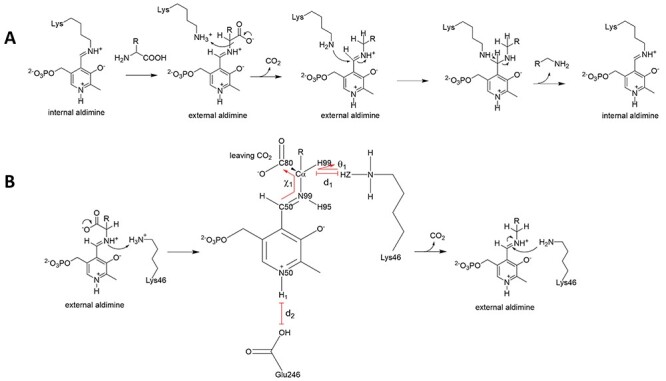
Schematic reaction mechanism of PLP-dependent decarboxylases and definition of reactive poses. (A) Upon entering the active site, the amino acid substrate replaces the enzyme bound lysine to form a new Schiff’s base structure with PLP, called the external aldimine. After decarboxylation, the internal aldimine is formed again with the same lysine residue and the decarboxylated product leaves the active site (Liang *et al.*, 2019). (B) Reactive pose with distance *d*_1_: H_Z_-C_α_; distance *d*_2_: O_E1_-H_1_; angle θ_1_: H_Z_-C_α_-H_99_ and dihedral χ_1_: C_50_-N_99_-C_α_-C_80_. The geometric parameters used to define reactive binding poses (*d*_1_, *d*_2_, θ_1_, χ_1_) reflect what happens during the C_α_-COO^−^ bond cleavage. The reaction requires the C_α_-COO^−^ bond be oriented such that the forming *p* orbital is aligned with the π orbitals of the Schiff base and the π system of the pyridine ring (χ_1_). Upon cleavage of the C_α_-COO^−^ bond, the nascent carbanion abstracts a proton from Lys46 (*d*_1_, θ_1_). The carbanion can be further stabilized by resonance delocalization in the PLP π system.

DAPDCs have been isolated and characterized from various organisms, including *M. tuberculosis*, *Arabidobsis thaliana*, *Helicobacter pylori* and *Methanococcus jannaschii* (Ray *et al.*, 2002; Gokulan *et al.*, 2003; Hu *et al.*, 2008; Crowther *et al.*, 2019). The structures of several DAPDCs have been solved by protein crystallography, including the enzymes from *Corynebacterium glutamaticum* (PDB 5X7N) (Son and Kim, 2018), *Escherichia coli* (1KNW), *H. pylori* (2QGH), *M. jannaschii* (1TWI) (Ray *et al.*, 2002) and *M. tuberculosis* (1HKW and 2O0T) (Gokulan *et al.*, 2003; Weyand *et al.*, 2009). The crystal structures provide information on the interactions of the substrate DAP with active site residues that govern substrate binding and on the catalytic mechanism (Momany *et al.*, 1995; Weyand *et al.*, 2009; Oliver *et al.*, 2014; Phillips *et al.*, 2019). Owing to the geometry of its active site pocket, DAPDC is able to distinctively bind the *meso*-stereoisomer of DAP with the D-stereocenter oriented close to the cofactor PLP where the amino group can react to form an external aldimine intermediate that undergoes decarboxylation. The L-stereocenter of DAP binds at the distal site of the substrate-binding region. The reaction initiates with the formation of an external aldimine in which the substrate’s amino group replaces the ε-NH_2_ of a conserved lysine that participates in an imine bond with the PLP cofactor when no substrate is present. For decarboxylation to occur, the leaving CO_2_ group at the D-binding site needs to be oriented in the direction away from the *Si*-face of C-4′ of the external aldimine (Hu *et al.*, 2008; Fogle and Toney, 2011). Decarboxylation is accompanied by protonation at the C_α_ carbon, followed by displacement of the substrate and reformation of the internal aldimine with the lysine (Fig. 1).

An attractive DAPDC for further exploration is the enzyme from the thermophilic bacterium *Thermotoga maritima* (*Tm*DAPDC). The enzyme has so far not been characterized but a crystal structure (PDB 2YXX) has been deposited. *Thermotoga maritima* is a hyperthermostable bacterium that dwells in hot springs and hydrothermal vents and is capable of growing at temperatures of up to 90°C (Nelson *et al.*, 1999). Consequently, *T. maritima* is expected to be a prolific source of thermostable enzymes. Enzyme thermostability facilitates engineering efforts and use in applied biocatalysis.

**Fig. 2:**
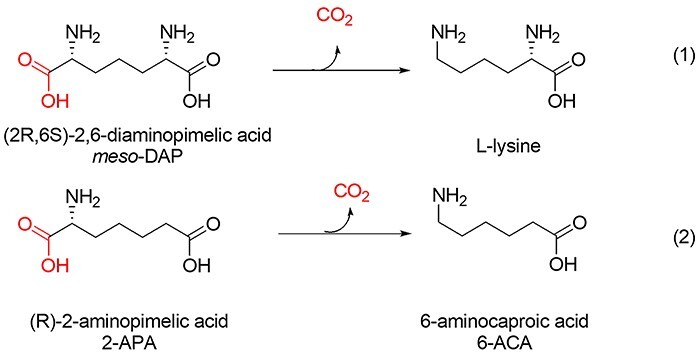
Decarboxylation reactions catalyzed by DAPDC. Diaminopimelic acid (DAP) is decarboxylated to L-lysine (1). Decarboxylation of 2-aminopimelic acid (2-APA) is of interest for engineering a biosynthetic pathway for 6-aminocaproic acid, an intermediate in nylon-6 production (2).

Our interest in DAPDCs was triggered by the possible application of a decarboxylase reaction in a metabolically engineered biosynthetic pathway toward the nylon-6 precursor 6-aminocaproic acid (6-ACA) (Turk *et al.*, 2016). In this artificial intracellular pathway in *E. coli*, the central metabolite 2-ketoglutarate is converted via a cascade of coexpressed heterologous proteins to 6-ACA. Side reactions within this pathway cause significant accumulation of 2-aminopimelic acid (2-APA), a compound which upon decarboxylation would give 6-ACA (Fig. 2). To explore the possibility of introducing *Tm*DAPDC into this pathway, we examined whether the selectivity can be tailored by protein engineering. Based on information derived from the X-ray structure, we rationally designed a small mutant library and tested the expressed mutant enzymes for decarboxylation of 2-APA under reaction conditions that might be relevant for 6-ACA production. To rationalize the observed decarboxylase activities of the mutant dataset with 2-APA and DAP, a combination of docking and MD simulations was performed. Computational analysis of geometric parameters of the mutant structures agreed that the best amino acids at position E315 for activity with 2-APA and DAP were Thr315 and Glu315, respectively.

## Materials and Methods

### Sequence and structure analysis

Sequences of enzymes homologous to DAPDC from *T. maritima* (encoded by the *lysA* gene, accession number Q9X1K5) were retrieved from the PDB database by Blast searches. A multiple sequence alignment of 10 related DAPDCs was constructed by T-Coffee and visualized by ESPript (Robert and Gouet, 2014). The crystal structure from the PDB entry 2YXX was compared with 10 related DAPDCs via the POSA server (Li *et al.*, 2014). The active site pocket was visualized with LigPlot (Laskowski and Swindells, 2011).

### Cloning of diaminopimelate decarboxylase

The codon optimized *lysA* gene, which encodes *Tm*DAPDC, was amplified by PCR (Table S1) to introduce an NcoI restriction site at the beginning and a polyhistidine tag coding sequence with an EcoRI restriction site at the end of the gene. The PCR product was introduced in the vector pBAD-MycHisA by restriction and ligation using T4 DNA ligase (Roche) under the recommended conditions. The ligation product was transformed into competent *E. coli* NEB-10β cells and grown on LB agar plates containing ampicillin (100 μg/ml). The plasmid was confirmed by sequencing (Eurofins Scientific) and used as the template for mutagenesis.

### Construction of the mutant library

Mutants were produced using the QuikChange site-directed mutagenesis protocol (Agilent). In a volume of 25 μl, 1 μl of forward and reverse primer (10-μM stock solution), 1-μl template plasmid (50–150 ng/μl stock concentration), 1.6% DMSO and 0.8-mM MgCl_2_ were mixed. Primers are given in Supporting Information (Table S1). After digestion with DpnI to remove template DNA, 5 μl of PCR product was transformed into competent *E. coli* NEB-10β cells and plated on LB agar containing 100-μg/ml ampicillin. The mutations in individual clones were confirmed by sequencing.

### Expression and purification of diaminopimelate decarboxylase

For the preculture, 30 ml of TB medium with 100-μg/ml ampicillin was inoculated with one colony of *E. coli* NEB-10β cells containing the pBAD-MycHisA-lysA plasmid and incubated overnight at 37°C. The preculture was diluted to an optical density OD_600_ of 0.6 in 200-ml TB medium with 100-μg/ml ampicillin and expression was started by adding 0.4% of L-arabinose. This main culture was incubated overnight at 37°C and harvested by centrifugation at 3000 × g and 4°C for 20–30 min. The cell pellet was resuspended in lysis buffer (20-mM TEA-HCl, pH 7.8, 10-mM imidazole, 50-μM PLP, 1-mM β-mercaptoethanol) with 1-mg/ml lysozyme and DNaseI (Sigma). To break the cells, the suspension was first incubated for 1 h at 37°C with shaking and then sonicated. Cell debris was removed by centrifugation (31 000 × g) to obtain the cell-free extract (CFE).

For immobilized metal ion affinity chromatography of His-tagged proteins, CFE was incubated overnight at 4°C with equilibrated Ni-NTA resin (Qiagen) under head-to-tail rotation. The incubated resin was extensively washed with wash buffer (lysis buffer with 20-mM imidazole and 300-mM KCl) and the His-tagged protein was eluted with a high concentration of imidazole (wash buffer with 300-mM imidazole). The eluted material was transferred onto an Amicon centrifugal unit with a MW cut-off of 30 kDa to concentrate the protein and exchange the buffer (50-mM TEA-HCl, pH 7.8, 20-μM PLP, 1-mM DTT, 50-mM KCl). Protein concentrations were determined using the Bradford assay and purity was confirmed by SDS-PAGE using 12% acrylamide gels.

### Spectrophotometric decarboxylation assays

To determine the activities of the wild-type enzyme, a coupled spectrophotometric enzyme assay was used (Diazyme Carbon Dioxide Enzymatic Assay Kit). In this assay, CO_2_ production is coupled to two enzymatic reactions. First, CO_2_ as bicarbonate reacts with phosphoenolpyruvate (PEP) to form oxaloacetate in a reaction catalyzed by PEP carboxylase. Next, oxaloacetate is reduced to malate by malate dehydrogenase with simultaneous oxidation of NADH to NAD^+^. The latter is followed spectrophotometrically at 340 nm. For the decarboxylase assays, 90 μl of the Diazyme kit solution was incubated with 100-μl substrate solution containing either DAP or 2-APA (Sigma-Aldrich) for 30 min at 30°C. Substrate stock solutions were prepared in reaction buffer (100-mM TEA-HCl, 20-μl PLP, 10-mM MgCl_2_) and were adjusted to pH 7.8. The stock solution for *rac*-APA was 40 mM, which equals the solubility limit. In order to reduce the CO_2_ content in assay mixtures, solutions were flushed with argon for 30 min prior to use. To start a reaction, 10 μl of wild-type enzyme solution of varying concentrations (1 mg/ml for the reactions with *meso*-DAP, 5 mg/ml for reactions with *rac*-APA) was added and the depletion of NADH was followed in the BioTek Synergy MX plate reader. Kinetic parameters were calculated from initial rates using the extinction coefficient of NADH at 340 nm, which is 6.22 mM^−1^·cm^−1^. The kinetic parameters for the substrate DAP were determined by varying substrate concentrations from 0 to 10 mM. The activities for 2-APA were determined at a concentration of 20 mM.

The activity profile of mutants of the E315X library was examined by following the depletion of NADH as consequence of CO_2_ production under similar conditions as mentioned above for assays with wild-type enzyme. Measurements were done with the standard substrates DAP or 2-APA at 10 or 20 mM, respectively. Kinetic parameters of the E315T mutant were determined by varying 2-APA concentrations from 0 to 10 mM. Measurements of enzymes from the E315X library with other substrates were also done at a substrate concentration of 20 mM.

### Thermostability

The thermostability of *Tm*DAPDC mutants was analyzed using the ThermoFluor assay (Ericsson *et al.*, 2006) in which the temperature-driven unfolding of a protein is detected by following the increase of fluorescence of Sypro Orange (Molecular Probes, Life Technologies). An enzyme solution (1 mg/ml) was mixed with 100× diluted Sypro Orange dye and heated from 20 to 99°C with a heating rate of 1.75°C/min in a CFX 96 Real Time PCR system (Bio-Rad). Fluorescence was followed with an excitation wavelength of 490 nm and an emission wavelength of 575 nm. Apparent melting temperatures were determined from the derivative of the fluorescence signal vs. temperature.

### Computational modeling

The external aldimine intermediate structures of DAP and 2-APA were prepared using YASARA (Krieger *et al.*, 2002) by adding a covalent bond between the substrate’s amino group and the exocyclic C4’ of PLP, followed up by an energy minimization using the GAFF force field (Wang *et al.*, 2004). A rotamer library of the external aldimine structures was created before Rosetta docking. First, the geometry of the energy-minimized external aldimine structures was further optimized in YASARA with the built-in QM-module using the semi-empirical AM1 method and the COSMO implicit solvent model (Klamt, 1995). The partial charges were derived using the AM1/BCC procedure (Jakalian *et al.*, 2002). The dihedral corresponding to the angle between the bond to the carboxylate group and the plane of the Schiff base (χ_1_) was set to −90°. A library containing 10 000 rotamers for both DAP and 2-APA was constructed by randomly perturbing the external aldimine heavy atom dihedrals while keeping atoms of the PLP moiety fixed. The rotamer library was subsequently pruned by: (i) energy criteria, (ii) uniqueness (RMSD > 0.0050 Å) and (iii) a cutoff (<2.5 Å) on the distance by which the distal C_α_ could deviate from a reference rotamer. The last pruning criterion was added to prevent sampling of irrelevant structures, and the reference rotamer was obtained by docking DAP into the wild-type DAPDC. After pruning, a final count of 400 unique rotamers remained in the DAP and 2-APA libraries (Fig. S2). The rotamer libraries were used as input for the Rosetta 2015 enzyme design application (Richter *et al.*, 2011), where the ligand was docked to the active site of DAPDC and E315X mutants were generated in a single dock-and-design step. Residues whose C_α_ or C_β_ were less than 8 or 10 Å, respectively, away from any ligand heavy atoms were set to repackable. For each E315X mutant, 500 output models were generated by Rosetta. Based on the Rosetta Interface Energy, the top-scoring scoring structures (50 out of 500) were selected as starting conformations for the MD simulations.

MD simulations were performed on the docking complexes containing the external aldimine of DAP or 2-APA to rank the structures. All MD simulations were run with YASARA (www.yasara.org) using the Yamber3 force field (Krieger *et al.*, 2004). The enzyme-ligand complex was placed in a dodecahedral cell 7.5 Å larger than the enzyme; 24 000 TIP3P waters were added to the cell, along with Na^+^ and Cl^−^ ions to a final concentration of 0.15 M. The system was then minimized with 1000 steps of steepest descent minimization, followed by 100 steps of simulated annealing. The system was gradually warmed up from 0 to 298 K during 3 ps and equilibrated for 2 ps. A multiple time step algorithm with a simulation time step of 1.25 fs was used (Krieger and Vriend, 2015). Long-range electrostatics were handled with the Particle Mesh Ewald algorithm at a cutoff distance of 7.86 Å (Essmann *et al.*, 1995). Temperature control was achieved using a modified Berendsen thermostat (Krieger *et al.*, 2004). Pressure was maintained with a Berendsen barostat (Berendsen *et al.*, 1984). Relevant distances, angles and dihedrals were computed on-the-fly during the 20-ps production run, and the trajectories were ranked by counting the number of reactive binding poses produced.

## Results

### Structure analysis

**Fig. 3:**
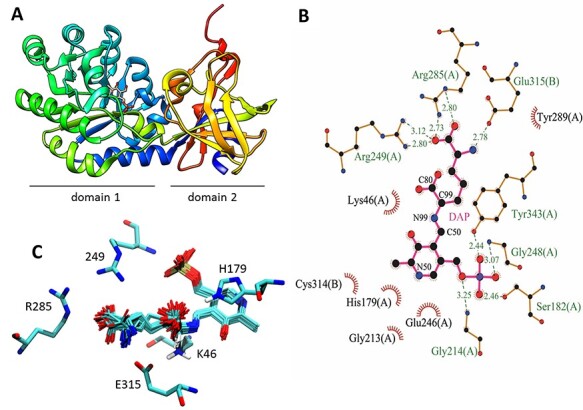
3D structure of the *Tm*DAPDC monomer. (A) Domain 1 is composed of an (α/β)_8_-TIM barrel and contains the active site with bound PLP. The N- and C-terminal regions of the protein (blue and yellow-red) form domain 2, mainly consisting of β-sheets. (B) 2D depiction of the DAP-bound active site. The iminium groups of Arg249 and Arg285 form salt bridges with the distal carboxylate, and Glu315 stabilizes the amino group of DAP. Created with LigPlot (Laskowski and Swindells, 2011). (C) Active site of *Tm*DAPDC with 50 docked structures of the external aldimine of 1DAP. The iminium groups of Arg249 and Arg285 form salt bridges to the distal carboxylate and Glu315 stabilize the amino group of *meso-*DAP.

To obtain a diaminopimelate decarboxylase with modified selectivity and activity with 2-APA, we chose as the starting point the diaminopimelate decarboxylase from the hyperthermophilic bacterium *T. maritima* (*Tm*DAPDC) of which the structure was solved by Nakamura and coworkers (PDB 2YXX). The enzyme has a dimeric structure in which the C-terminus of one monomer is wrapped around the other (Fig. S3), a topology that is conserved in related PLP-dependent fold-type III enzymes (Crowther *et al.*, 2019). While the overall structure is highly conserved, sequence similarities between *Tm*DAPDC and other DAPDCs are modest (<36%) (Table S2). When aligning the structures of *Tm*DAPDC with 10 other DAPDCs using the POSA server (Li *et al.*, 2014), the average RMSD was 1.70 Å (319 equivalent α-carbons) making the structures quite similar (Fig. S4). Each monomer consists of a large (α/β)_8_ TIM-barrel like domain and a smaller domain composed of a β-sandwich and short α-helices. The small domain is formed by the C- and N-terminal continuation of the first domain (Fig. 3A and B). The active site is buried in a cleft between the two domains. It flanks the TIM barrel at the monomer–monomer interface and is partially solvent exposed. An active site lid contributed by the second monomer (residues 133–152) closes the active site upon substrate binding and plays a role in substrate recognition at the reactive D-carboxylate binding site (Hu *et al.*, 2008; Phillips *et al.*, 2019).

The alignment also shows that the substrate binding site is highly conserved among DAPDCs (Fig. S5). Residues Tyr343 and Ser182 are involved in PLP binding through the phosphate group. Glu246 and Asp65 keep the pyridinium nitrogen of PLP protonated to facilitate its function as electron sink (Eliot and Kirsch, 2004). His179 interacts with the PLP ring via π-stacking. The conserved Lys46 is bound to PLP in the substrate-free enzyme, forming the internal aldimine. After substrate binding, formation of the external aldimine and cleavage of the C_α_-CO_2_ bond, the lysine will replace the PLP-bound product. When Lys46 is free, it can form a salt bridge with Asp65 (Hu *et al.*, 2008). The flexibility of Lys46 thus is important in the catalytic cycle of the enzyme. In the external aldimine, the carboxyl group that is cleaved off will be oriented at the *Si*-face of C4’ carbon of the PLP, as described for *M. jannaschii* DAPDC (Ray *et al.*, 2002; Fogle and Toney, 2011). The leaving CO_2_ is on the opposite side of Lys46. In addition to the reactive site, there is a distinct binding site for the distal or non-reacting L-amino acid part of DAP (Ray *et al.*, 2002).

To examine possible binding conformations of the external aldimine that undergoes decarboxylation, we performed docking simulations with different rotamers of PLP-bound DAP (Fig. 3C). The results indicated that for different aldimine conformations, the iminium groups of Arg249 and Arg285 can stabilize the distal (non-reacting) carboxylate group of DAP, whereas Glu315, belonging to the other subunit, is important for the recognition of the non-reacting ε-amino group. Glu315 was therefore chosen as the target for mutagenesis, since its replacement is expected to modify the selectivity of the enzyme for the distal group, including the possible introduction of activity with 2-APA, which lacks the distal ε-amino group (Fig. 2).

### Catalytic properties of E315 mutants

**Fig. 4:**
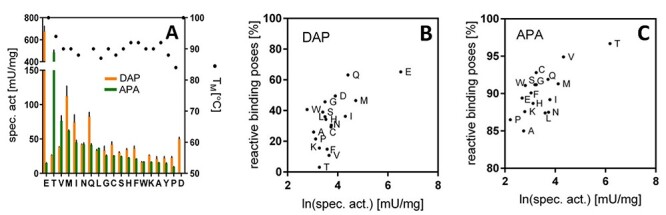
Experimental and predicted activities of the E315X library variants for activities with DAP and *rac*-APA. (A) Experimental activities (orange bars, *meso*-DAP; green bars, 2-APA) and thermal stabilities (T_M_). (B and C) Correlation between reactive binding poses observed in MD simulations and catalytic activity of E315X DAPDC mutants for *meso*-DAP and 2-APA, respectively. For *meso*-DAP, the geometric parameters for defining reactive binding poses were *d*_1_ < 4.0 Å, θ_1_ < 80°, −90° < χ_1_ < −120°, *d*_2_ < 2.0 Å. The Pearson correlation is *r* = 0.63. Giving the larger proportion of reactive binding poses, the wild-type DAPDC (E315E) was thus correctly identified as the best variant for *meso*-DAP. For 2-APA, the parameters were *d*_1_ < 4.0 Å, θ_1_ < 80°, −75° < χ_1_ < −125°, *d*_2_ < 2.0 Å. The Pearson correlation is *r* = 0.72. Mutant E315T was correctly identified as the best variant for 2-APA.

To examine the possibility to introduce new activities, a set of mutants was created in which Glu315 was replaced by the other 19 proteinogenic amino acids. All *Tm*DAPDC mutants were constructed individually by QuikChange PCR and were well expressed in *E. coli* with yields reaching 0.5–1 mg/ml culture. Measurements of apparent melting temperatures of enzymes purified by His-tag affinity chromatography (Fig. S6) revealed that all mutants could be isolated although some variants showed some reduction of stability (Δ*T*_m,app_ < 10°C) (Fig. 4A). The mutant enzymes were examined for activities toward DAP and 2-APA. Most of the mutants retained activity toward the natural substrate DAP, although significantly lower than the wild-type (Fig. 4A). Most mutants also gained activity toward 2-APA, which was very low with wild-type DAPDC, confirming that Glu315 is important for recognition of the distal amino group of DAP.

Mutant E315T is particularly interesting since it has lost most of the activity toward DAP and has the highest activity toward 2-APA. Activity was higher with 2-APA than with DAP. The inversion of substrate preference toward 2-APA was also apparent from the kinetic parameters (Table I). Whereas both wild-type DAPDC and the E315T mutant showed similar *K_M_* values with DAP and 2-APA (around 0.3 mM), the *k*_cat_ of the mutant with 2-APA was 2.6-fold higher than that of the wild-type with DAP. This suggests that weaker substrate binding expected for 2-APA in wild-type DAPDC indeed is avoided by the E315T mutation, allowing good binding of DAP and 2-APA in the wild-type and mutant, respectively. Furthermore, the E315T mutant fully retained the enzyme stereopreference by only decarboxylating D-APA present in a racemic mixture (Fig. S7).

**Table I:** Kinetic parameters of *Tm*DAPDC variants

Enzyme	Substrate	K_M_ (mM)	*k*_cat_ (s^−1^)
Wild-type	DAP	0.27 (0.06)	0.075 (0.004)
E315T	APA	0.32 (0.03)	0.2 (0.004)

Standard deviations from triplicate measurements are given in parenthesis.

### Substrate scope and promiscuity

Because substitutions of E315 introduced activity with 2-APA in DAPDC, we examined the activity of the E315X mutants also with other D-amino acids. A broad substrate range might identify a versatile D-amino acid decarboxylase, adding to the portfolio of enzymes acting on D-stereocenters, which are not as widespread in nature as enzymes acting on L-amino acids. DAPDCs are the only known decarboxylases that can remove a carboxyl group from a D-stereocenter. We tested eight compounds, differing in side-chain length and distal group functionality (Fig. S8, Table II). To explain shifts in activity, models of variant E315T with docked external aldimine intermediate of the substrates were generated (Fig. S9).

**Table II:** Activities of mutants of *Tm*DAPDC with different substrates

Substrate/mutant	Specific activity (mU/mg)							
	WT	E315T	E315F	E315P	E315M	E315L	E315Q	E315N
*meso*-DAP^c^	675 (70)	26.3 (0.9)	36.0 (6.5)	22.6 (3.4)	111 (21)	32.7 (3.9)	82 (11)	42 (1)
2-APA^c^	14.7 (1.5)	482 (36)	21 (1)	9.1 (1.5)	62 (3)	36.5 (0.7)	41 (4)	41.6 (5)
α-aminoadipic acid^c^	n.a.^a^	3.2 (0.2)	3.2 (0.7)	7.3 (0.4)	13 (2)	12.4 (0.8)	8.1 (0.6)	32.3 (2.6)
D-glutamate	n.a.	n.a.	n.a.	n.a.	n.a.	n.a.	5.9 (0.1)	n.a.
D-ornithine	26.6 (4.3)	n.a.	n.a.	n.a.	n.a.	n.a.	n.a.	n.a.
D-norleucine	n.a.	n.a.	4.2 (0.2)	n.a.	n.a.	13.8 (0.4)	n.a.	n.a.
D-phenylalanine	n.a.	n.a.	4.1 (0.1)	n.a.	n.a.	13.3 (0.9)	n.a.	n.a.

^a^n.a., No activity detectable (i.e. <1 mU/mg). L- and D-lysine were also tested but no activity was found with any of the mutants.

^b^Errors (standard deviations of triplicate measurements) in parenthesis.

^c^Mixture of enantiomers.

The results confirm that the amino acid at position E315 strongly influences substrate range. The only DAPDC variant that showed activity toward D-ornithine was WT, and docking indicated that its distal amino group can form a salt bridge with E315 (Fig. S9A). While in variant E315T, the distal carboxylate group of meso-DAP and 2-APA can form salt bridges with residues R285 and R249, D-ornithine obviously cannot do so in mutants lacking E315 and activity was only observed with the wild-type. The related ODCs and arginine decarboxylases have an aspartate at position E315 for salt-bridge interaction with the respective side-chain amino groups (Osterman *et al.*, 1995; Hu *et al.*, 2008).

For the three substrates that only contain a carboxylate in the side chain, the enzymatic activity decreases as the side chain length decreases, i.e., activity was higher with 2-APA than with α-aminoadipic acid and D-glutamate. A shorter side chain in the substrate hinders the ability of the distal carboxylate to form salt bridge interactions with R285 and R249 (Fig. S9B). On the other hand, a hydrophobic group at the position of E315 is required for conversion of D-amino acids with a hydrophobic side chain (D-phenylalanine and D-norleucine), for which no detectable activity was found in other E315X variants. L-lysine, the product of the natural DAP decarboxylation, was not reactive in E315X variants and neither was the enantiomeric D-lysine.

### Analysis of decarboxylase activity by MD simulations

To examine if the selectivity of DAPDC variants with DAP and 2-APA can be computationally predicted, we modeled enzyme-substrate complexes relevant for the decarboxylation reaction. The external aldimine intermediate was chosen for modeling because the C_α_-COO^−^ bond cleavage takes place on this intermediate (Fig. 1A), and in PLP-dependent enzymatic reactions, the step prior to the formation of the quinonoid intermediate is typically rate-limiting (Zhou *et al.*, 2001; Toney, 2005). Using a combination of docking and molecular dynamics simulations, we analyzed binding poses of the external aldimine that could reveal geometric factors correlated with the observed experimental activities.

The enzyme-ligand complexes were obtained by covalent docking of the external aldimine of different substrates into the active site using Rosetta (Fig. S10). The resulting structures (500 per enzyme-ligand complex) were examined prior to the MD simulations. A trend emerges when comparing the averaged Rosetta Interface Energies (500 structures for each E315X mutant) to the experimental catalytic activities (Table S3). Interface Energies are given in Rosetta Energy Units and measure the interaction between the ligand and the rest of the protein. Mutants with higher specific activities generally had a lower Interface Energy, which indicated that the enzyme could more easily accommodate the external aldimine. The best mutant for 2-APA (E315T) is also the best mutant in terms of the docking score. Wild-type DAPDC is correctly predicted to have a low activity toward 2-APA. On the other hand, docking scores of the external aldimine intermediate of DAP show worse Interface Energies in wild-type DAPDC than expected. E315M is correctly identified to be a good mutant. 

The subsequent multiple short MD simulations (20-ps runs) were aimed at determining the frequency of occurrence of reactive poses. Based on the reaction mechanism, several geometric criteria required for decarboxylation to occur can be postulated (Fig. 1B): (i) the extraction angle between the cleavable sigma bond (C_α_-COO^−^) and the flat π system of the PLP ring (χ_1_), (ii) the proximity of the catalytic residue Lys46 to the C_α_ carbon (*d*_1_, θ_1_) and (iii) the interaction between the PLP cofactor and the protein environment as reflected in the distance between the pyridine nitrogen and Glu246 (*d*_2_). The trajectories were ranked by counting the number of frames with reactive binding poses. For both DAP (Fig. 4B) and 2-APA (Fig. 4C), the most reactive mutants were found to have a larger proportion of reactive binding poses than the least reactive mutants, indicating that the combination of Rosetta docking and MD can explain the trend in activity ratios and identify the best mutant for (*R*)-2-APA decarboxylation.

The total number of reactive binding poses was governed by the conformation of the catalytic Lys46 (*d*_1_, θ_1_), and not the orientation of the extracted COO^−^ (χ_1_) or the cofactor’s position in the binding pocket (*d*_2_). During the short 20-ps trajectories, the χ_1_ dihedral did not move away from the initial value set at the docking stage, remaining close to −90° and steadily producing reactive binding poses throughout the simulation. Furthermore, the distance from the pyridinium hydrogen (H_1_) to Glu246 (*d*_2_) had an autocorrelation time (*t* < 0.1 ps) much shorter than the total simulation time, going in and out of the reactive pose (*d*_2_ < 2.0 Å) hundreds of times during the trajectory. However, both criteria (χ_1_ and *d*_2_) were kept in the definition of reactive poses for consistency.

A closer examination of the conformations of the catalytic Lys46 revealed it adopted one of three distinct conformations during docking and in the subsequent MD simulations (conformations MC1-MC3) (Fig. S11). These conformations rarely interchanged between each other in the 20-ps trajectories. In MC1, Lys-NH_3_^+^ is stabilized by interactions with the O connected to the PLP ring, sitting in-between Tyr343A (-OH) and Tyr351B (-OH). This conformation has shorter *d*_1_ distances (average 2.9 Å), thereby producing more reactive binding poses than conformations MC2 and MC3. Conformation MC3 was the least productive, with a distance *d*_1_ > 4.8 Å it rarely met the *d*_1_ criterion (*d*_1_ < 4.0 Å). In MC2, Lys-NH_3_^+^ moves to the left and has interactions with Asp65A (side chain). In MC3, Lys-NH_3_^+^ has moved further away, displacing one water molecule and occupying its place, making interactions with Asp65A (side chain), Asp65A (backbone) and Glu71A. Consequently, the MD simulations suggest that MC2 and MC3 represent local minima which are less reactive than MC1.

## Discussion

PLP-dependent diaminopimelate decarboxylases act on the (*R*)-D-stereocenter of diaminopimelate and have a high substrate specificity, related to their crucial role in lysine biosynthesis. The enzyme from *T. maritima*, *Tm*DAPDC, also shows a narrow substrate range, although there is some promiscuity by accepting 2-APA and D-ornithine along with DAP. The somewhat relaxed selectivity of the wild-type *Tm*DAPDC may be related to *T. maritima* being positioned in one of the slowest evolving branches of eubacteria (Nelson *et al.*, 1999). The organism appears to have a high percentage of archaeal genes. Within the family of fold-type III PLP enzymes, DAPDCs are more similar to ODCs than to arginine decarboxylases, which may relate to the observed low activity of *Tm*DAPDC with D-ornithine (Kidron *et al.*, 2007). Ray *et al.* (2002) postulated that during the evolution of this structural family, an internal ‘molecular ruler’ developed in the active site to accept amino acid side chains with increasing length and chemical complexity, from alanine racemase to ODC and DAPDC. From structure alignments of the active sites, it appeared that the position of the residue interacting with the distal charged group varies with the length of the substrate side chain to allow salt bridge formation. For binding of alanine, only interactions close to the PLP binding site are important, whereas for ODC and DAPDC, interactions at a more distal binding site contribute to substrate recognition as well. The distal amino group of ODC from *Trypanosoma brucei* is recognized via a salt bridge with Asp361 located on the opposite subunit. In *Tm*DAPC, the distal amino group recognition is undertaken by residue Glu315, which is also positioned in the other subunit. Accordingly, we found that replacements at position Glu315 did not only disturb activity with the native substrate DAP but also expanded the substrate scope by removing this molecular ruler.

To our knowledge, there are no reports describing engineering of bacterial DAPDCs aimed at developing biotechnological applications. The engineering of D-amino acid decarboxylase variants with enhanced or shifted substrate scope could open up new possibilities for use of D-amino acids as intermediates in engineered biosynthetic pathways since this would allow routes to operate orthogonal to the central L-amino acid metabolism of a host organism. For example, decarboxylation of D-2-APA would yield the nylon-6 precursor 6-aminocaproic acid. Motivated to find a mutant enzyme that is active with 2-APA, we picked Glu315 as starting point for mutagenesis and tried to explain the resulting activities with computational methods. Under conditions postulated for *in vivo* application, the E315T mutant showed almost opposite relative activities toward DAP and 2-APA compared with the wild-type enzyme, indicating that catalytic activity indeed is not only determined by the positioning of the carboxylate at the proximal (reactive) substrate binding site (Fogle and Toney, 2011), but also by interactions at the distal L-binding site. In addition to being a catalytically efficient enzyme, the E315T variant also shows excellent enantioselectivity toward D-2-APA. We are currently examining the possibility of integrating the activity of the *Tm*DAPDC E315T mutant in an alternative biosynthetic pathway toward the production of 6-ACA (Turk *et al.*, 2016), with D-2-APA as intermediate.

The decarboxylation products of lysine and ornithine are cadaverine and putrescine, respectively, which are used in polymer chemistry and in pharmaceutical applications (Schneider and Wendisch, 2011). Cadaverine is also the main precursor for a variety of quinolizidine alkaloids-based pharmaceutical compounds (Yamazaki *et al.*, 2012). In natural pathways, these amines are formed by decarboxylation of L-amino acid precursors. The shorter analog of 6-aminocaproic acid, 5-aminovaleric acid, is a precursor for valerolactam, which is used in nylon-5 synthesis and can be reached by decarboxylation of 2-aminoadipic acid, which is formed in fungi as an intermediate in antibiotic biosynthesis. The bulkier substrate D-phenylalanine was accepted by the *Tm*DAPDC mutants E315F and E315L. Various other *Tm*DAPDC mutants were discovered with different substrate scope than the wild-type, but the observed activities are still low. Whereas there are alternative methods to obtain some of the products, the observation that the specificity of *Tm*DAPDC can be altered by mutagenesis suggests that the enzyme is an attractive template for redesign toward biocatalytic applications.

Computational modeling of the *Tm*DAPDC mutants containing the external aldimine intermediates revealed a modest correlation between the proportion of frames with reactive binding poses and experimental activity. The modesty of the correlation suggests that the geometric description of the decarboxylation used in this study (Fig. 1B) may not capture the final decarboxylation step accurately (Fig. 1A). First, it is unknown whether decarboxylation by DAPDC is a concerted process, where loss of CO_2_ and protonation of the C_α_ occur in the same step (Fogle and Toney, 2011; Phillips *et al.*, 2019). Were it not a concerted process, Lys46-NH_3_^+^ would not be needed in close proximity to C_α_ (distance *d*_1_) at the exact same instant when the CO_2_ loss occurs. The observed correlation (Fig. 4) leans toward a concerted process, in line with evidence from deuterium isotope effect experiments that the electrophilic substitution occurs at the same time of CO_2_ loss or immediately after (Kelland *et al.*, 1985). Second, the identity of the general acid that protonates C_α_ is still unknown but believed to be Lys46, which would be consistent with the stereoselectivity of the reaction and with our observations (Jackson *et al.*, 2000; Fogle and Toney, 2011; Phillips *et al.*, 2019). Third, it is uncertain to what extent the distance from the pyridinium H_1_ and Glu246 (*d*_2_) can influence the enzymatic activity. A non-concerted process would particularly benefit from the pyridine’s ability to distribute the developing negative charge of the quinonoid intermediate during C_α_-CO_2_ cleavage. Studies on dialkylglycine decarboxylase have shown that the pyridine ring may only play a small role in the stabilization of C_4_’ carbanions, with most of the stereoelectronic stabilization coming from the Schiff base (Bach *et al.*, 1999; Toney, 2001).

A previous study (Fogle and Toney, 2011) found a weak correlation between the optimal χ_1_ dihedral and the experimental activity of a DAPDC from *M. tuberculosis* with several substrates. In contrast, we worked on the assumption that all mutants can position the external aldimine in a catalytic conformation (χ_1_ = −90°) and tested how easily that conformation can be maintained (χ_1_ criterion) during the 20-ps MD trajectory while keeping the ligand in a reactive binding pose (θ_1_, *d*_1_ and *d*_2_ criteria). The approach enabled the identification of the best enzyme variants by counting the number of frames representing reactive binding poses. Similarly, the docking scores of DAP and 2-APA show a correlation to experimental activities (Table S3). E315X mutants with higher activities have better docking scores, meaning that it is easier for Rosetta to accommodate the intermediate in the binding pocket. Because substrate binding is most likely not rate-limiting, the binding kinetics of the substrates was not considered in this study, leaving out the possibility that the E315X mutations interfere in reaction steps prior to the external aldimine formation.

PLP is a versatile cofactor enabling a variety of chemical reactions on amino acids, such as transamination, racemization, β- and γ-elimination, aldol cleavage and decarboxylation. The type of reaction is determined by the geometry of active site residues and the interactions between substrate, enzyme and cofactor (Hu *et al.*, 2008; Phillips *et al.*, 2019). Applying predictive methods to PLP-dependent enzymes is difficult due to the multistep catalytic cycle. Yet, computation-supported engineering of class III transaminases has been achieved (Seo *et al.*, 2012; Sirin *et al.*, 2014; Han *et al.*, 2017; Voss *et al.*, 2018). Studies on computational modeling of α-amino acid decarboxylases are less abundant, despite having a simpler reaction mechanism in the sense that only one substrate is involved in the reaction cycle. The ability to computationally explain the reactivities and selectivities of sets of mutants of such decarboxylases would allow design of useful variants in a single step, avoiding the necessity to test large libraries with multiple iterations of mutagenesis and testing.

## Supplementary Material

AM_Decarboxylase_Supporting_R1_gzab016
