# Supplementary material for: Thermostable D-amino acid decarboxylases derived from Thermotoga maritima diaminopimelate decarboxylase
Source: Protein Eng Des Sel. 2021 Jul 13;34:gzab016. doi: 10.1093/protein/gzab016 (PMC8277567; doi:10.1093/protein/gzab016)
Supplement: AM_Decarboxylase_Supporting_R1_gzab016 [file am_decarboxylase_supporting_r1_gzab016.pdf]

Supporting Information

Thermostable D-amino acid decarboxylases derived from *Thermotoga maritima* diaminopimelate decarboxylase

Antonija Marjanovic[1], Carlos Ramírez-Palacios [1,2], Marcelo F. Masman[1,2,3], Jeroen Drenth[1], Marleen Otzen[1], Siewert-Jan Marrink[2], Dick B. Janssen[1*]

[1]Biotechnology and Biocatalysis and [2]Molecular Dynamics Group, Groningen Biomolecular Sciences and Biotechnology Institute, University of Groningen, The Netherlands

[3]Present address: Van't Hoff Institute for Molecular Sciences HIMS-Biocat, University of Amsterdam, The Netherlands

[*] Corresponding author, e-mail: d.b.janssen@rug.nl

Table S1: Oligonucleotides used for cloning

Oligonucleotide	Oligonucleotide sequence (5' to 3')
389° LysATma-F	AACCCCATGGACATCCTGAGAAAGG
389° LysATma-H6-R	CGCCACCACTTTGTACAAGAATTCTTAGTGATGGTGATGATGATGCATAACCACATCC
TmaH6 E315F° F	CCCTCTGTGCtttAGCGGTGATGTTATTGCTTACG
TmaH6 E315F° R	CGTAAGCAATAACATCACCGCTaaaGCACAGAGGG
TmaH6 E315L° F	CCCTCTGTGCctgAGCGGTGATGTTATTGCTTACG
TmaH6 E315L° R	CGTAAGCAATAACATCACCGCTcagGCACAGAGGG
TmaH6 E315I° F	CCCTCTGTGCattAGCGGTGATGTTATTGCTTACG
TmaH6 E315I° R	CGTAAGCAATAACATCACCGCTaatGCACAGAGGG
TmaH6 E315V° F	CCCTCTGTGCgtgAGCGGTGATGTTATTGCTTACG
TmaH6 E315V° R	CGTAAGCAATAACATCACCGCTcacGCACAGAGGG
TmaH6 E315M° F	CCCTCTGTGCatgAGCGGTGATGTTATTGCTTACG
TmaH6 E315M° R	CGTAAGCAATAACATCACCGCTcatGCACAGAGGG
TmaH6 E315P° F	CCCTCTGTGCccgAGCGGTGATGTTATTGCTTACG
TmaH6 E315P° R	CGTAAGCAATAACATCACCGCTcggGCACAGAGGG
TmaH6 E315T° F	CCCTCTGTGCaccAGCGGTGATGTTATTGCTTACG
TmaH6 E315T° R	CGTAAGCAATAACATCACCGCTggtGCACAGAGGG
TmaH6 E315A° F	CCCTCTGTGCgcgAGCGGTGATGTTATTGCTTACG
TmaH6 E315A° R	CGTAAGCAATAACATCACCGCTcgcGCACAGAGGG
TmaH6 E315Y° F	CCCTCTGTGCtatAGCGGTGATGTTATTGCTTACG
TmaH6 E315Y° R	CGTAAGCAATAACATCACCGCTataGCACAGAGGG
TmaH6 E315H° F	CCCTCTGTGCcatAGCGGTGATGTTATTGCTTACG

TmaH6 E315H° R	CGTAAGCAATAACATCACCGCTatgGCACAGAGGG
TmaH6 E315Q° F	CCCTCTGTGCcagAGCGGTGATGTTATTGCTTACG
TmaH6 E315Q° R	CGTAAGCAATAACATCACCGCTctgGCACAGAGGG
TmaH6 E315N° F	CCCTCTGTGCaacAGCGGTGATGTTATTGCTTACG
TmaH6 E315N° R	CGTAAGCAATAACATCACCGCTgttGCACAGAGGG
TmaH6 E315K° F	CCCTCTGTGCaaaAGCGGTGATGTTATTGCTTACG
TmaH6 E315K° R	CGTAAGCAATAACATCACCGCTtttGCACAGAGGG
TmaH6 E315D° F	CCCTCTGTGCgatAGCGGTGATGTTATTGCTTACG
TmaH6 E315D° R	CGTAAGCAATAACATCACCGCTatcGCACAGAGGG
TmaH6 E315C° F	CCCTCTGTGCtgcAGCGGTGATGTTATTGCTTACG
TmaH6 E315C° R	CGTAAGCAATAACATCACCGCTgcaGCACAGAGGG
TmaH6 E315W° F	CCCTCTGTGCtggAGCGGTGATGTTATTGCTTACG
TmaH6 E315W° R	CGTAAGCAATAACATCACCGCTccaGCACAGAGGG
TmaH6 E315R° F	CCCTCTGTGCcgtAGCGGTGATGTTATTGCTTACG
TmaH6 E315R° R	CGTAAGCAATAACATCACCGCTacgGCACAGAGGG
TmaH6 E315S° F	CCCTCTGTGCagcAGCGGTGATGTTATTGCTTACG
TmaH6 E315S° R	CGTAAGCAATAACATCACCGCTgctGCACAGAGGG
TmaH6 E315G° F	CCCTCTGTGCggcAGCGGTGATGTTATTGCTTACG
TmaH6 E315G° R	CGTAAGCAATAACATCACCGCTgccGCACAGAGGG

Table S2: Sequence identities (%) determined with the POSA server for DAPDCs with known crystal structure.

	2yxx<br>*T. ma*	6n2f<br>*A. th*	5x7m<br>*C. gl*	4xg1<br>*P .in*	2p3e<br>*A. ae*	1twi<br>*M.ja*	3n2b<br>*V. ch*	3vab<br>*B. me*	1hkv<br>*M. tu*	1knw<br>*E. co*	2qgh<br>*H. py*
2yxx	-	32.1	30.3	30.1	35.7	35.4	32.6	34.8	29.9	28.4	35.5
6n2f	32.1	-	25.2	30.8	33.8	30.1	32.1	32.4	24.2	25.5	31.7
5x7m	30.3	25.2	-	25.6	31.6	27.7	28.1	27.7	57.3	24.9	28.0
4xg1	30.1	30.8	25.6	-	44.4	36.9	56.2	44.7	24.4	27.1	39.9
2p3e	35.7	33.8	31.6	44.4	-	38.8	41.7	42.0	30.3	28.7	41.8
1twi	35.4	30.1	27.7	36.9	38.8	-	34.4	35.3	29.4	26.2	37.9
3n2b	32.6	32.1	28.1	56.2	41.7	34.4	-	48.3	26.0	26.3	42.2
3vab	34.8	32.4	27.7	44.7	42.0	35.3	48.3	-	31.9	29.1	41.7
1hkv	29.9	24.2	57.3	24.4	30.3	29.4	26.0	31.9	-	23.3	26.5
1knw	28.4	25.5	24.9	27.1	28.7	26.2	26.3	29.1	23.3	-	29.4
2qgh	35.5	31.7	28.0	39.9	41.8	37.9	42.2	41.7	26.5	29.4	-

Table S3. Comparison of the Interface Energies (REU) of E315X mutants obtained in the docking stage and the experimental activity.

2-APA				DAP		
Mutant	Activity (mU/mg)	Docking score (REU)		Mutant	Activity (mU/mg)	Docking score (REU)
E315T	482.2	-11.11		WT	674.9	-7.39
E315V	75.9	-11.06		E315M	112.2	-9.14
E315M	61.8	-10.96		E315Q	82.5	-8.66
E315I	44.4	-11.08		E315I	74.1	-8.91
E315N	41.7	-10.88		E315D	49.6	-8.61
E315Q	40.9	-10.66		E315N	42.1	-8.23
E315L	36.5	-10.55		E315C	41.7	-9.05
E315G	25.6	-11.00		E315V	38.4	-9.36
E315C	25.6	-11.10		E315F	36	-8.03
E315S	24.4	-10.99		E315H	34.2	-7.72
E315H	22.6	-10.05		E315L	32.7	-7.62
E315F	20.9	-10.51		E315G	32.9	-8.89
E315W	16.6	-9.71		E315S	29.9	-8.70
E315K	16.2	-10.27		E315T	26.3	-8.91
E315A	15.5	-11.24		E315K	26.3	-7.45
WT	14.7	-8.44		E315P	22.6	-8.61
E315P	9.1	-10.77		E315A	20.9	-8.72
				E315W	16.2	-7.12

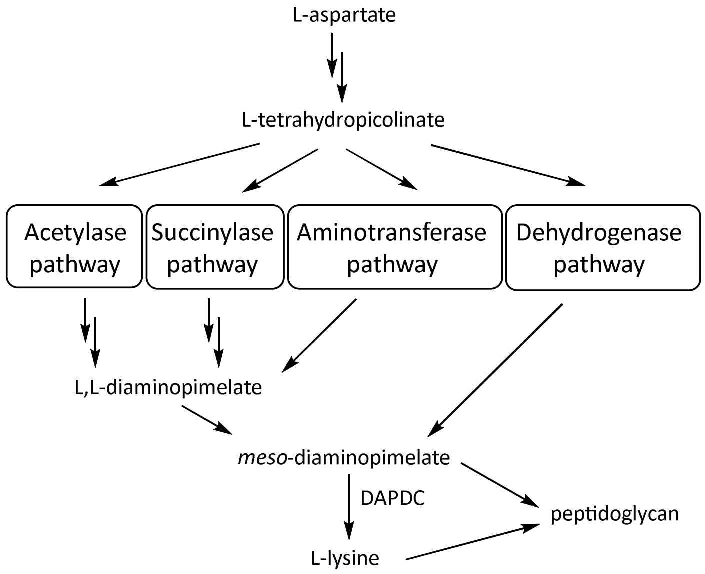

Figure S1: Four known anabolic L-lysine pathways. The acetylase and succinylase pathways share similar intermediates except for the incorporation of acetyl-CoA vs. succinyl-CoA, respectively. In the aminotransferase pathway, L-tetrahydropicolinate is converted to L,L-DAP by an aminotransferase (DapL). In the dehydrogenase pathway, L-tetrahydropicolinate is directly converted to DAP. All pathways share the last step, the conversion of DAP to L-Lys by DAPDC.

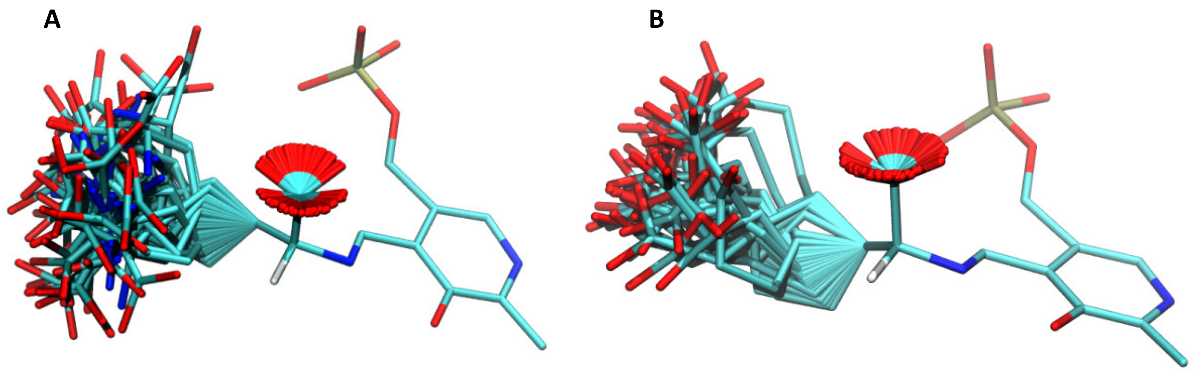

Figure S2: Rotamer libraries of PLP-bound substrates *meso*-DAP (**A**) and (*R*)-APA (**B**).

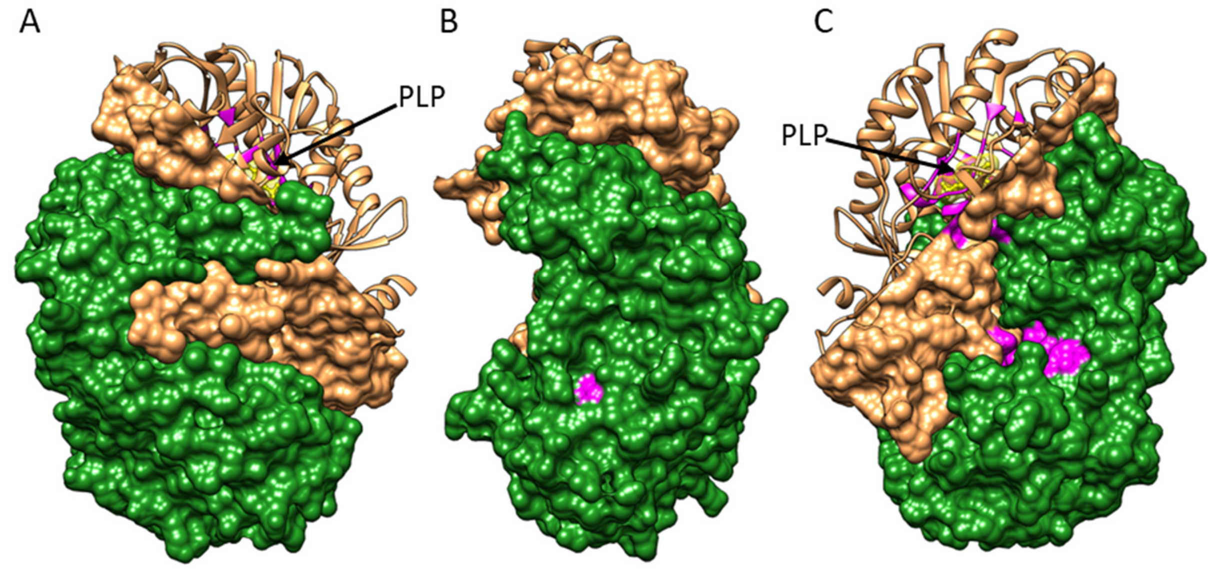

Figure S3: DAPDC dimer of *T. maritima* in different perspectives. **A**: C-terminus of one monomer wrapped around the other like two right handed mittens. **B**: Side perspective with a large water exposed area and a tunnel towards the active site. **C**: 180° rotation relative to panel A. The active site is water exposed Both monomers contribute to the active site geometry. PLP in yellow with active site in pink.

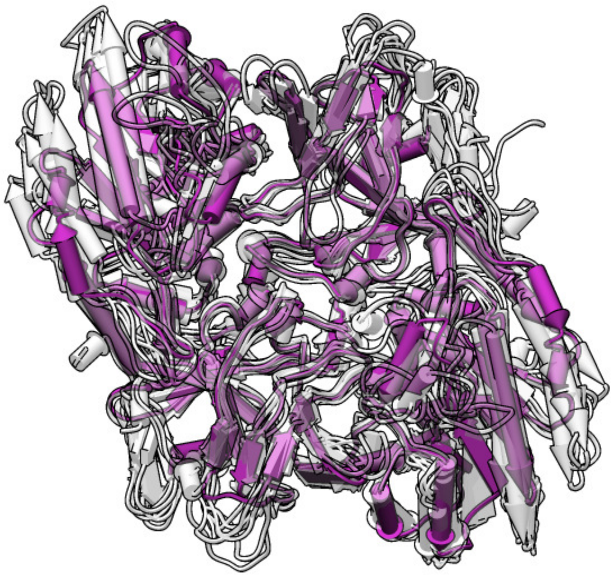

Figure S4: Structural alignment of DAPDCs. In opaque grey are the crystal structures of the following DAPDCs: 1KNW (*E. coli)*; 5X7M (*C. glutamicum*); 1HKV (*M. tuberculosis)*; 6N2F (*A. thaliana)*; 1TWI (*M. jannashii)*; 2QGH (*H. pylori)*; 2P3E (*A. aeolicus)*; 3VAB (*B. melitensis)*; 3N2B (*V. cholerae)*; 4XG1 (*P. ingrahamii)* and in purple the reference structure 2YXX (*T. maritima*). The secondary structure is highly conserved: RMSD 1.7Å.

```
                                                              α1             β1
pdb|2YXX|A                                                    1    10
pdb|2YXX|A  ..........................................XDILRKVAEIH.GTPTYV
pdb|1KNW|A  ...........................MPHSLFSTDTDLTA..ENLLRLPAEF.GCPVWV
pdb|5X7M|A  ............MATVENFNELPAHVWPRNAVRQEDGVVTVAGVPLPDLAEEY.GTPLFV
pdb|1HKV|A  ...............MNELLHLAPNVWPRNTTRDEVGVVCIAGIPLTQLAQEY.GTPLFV
pdb|6N2F|A  ..........................FDHCFKKSSDGFLYCEGTKVQDIMETVEKRPFYL
pdb|1TWI|A  .......................M.LGNDTVEI.KDGRFFIDGYDAIELAEKF.GTPLYV
pdb|2QGH|A  ........MGSSHHHHHHSSGLVP.RGSHMF.............NYEELFQTH.KTPFYL
pdb|2P3E|A  ...................MELLK.EYNPYLEYR.DGELFIEGVSLKELAQTF.GTPLYV
pdb|3VAB|A  ....MAHHHHHHMGTLEAQTQGPG.SMVNHFEYR.NGVLHAENVSLPEIAKAV.GTPFYV
pdb|3N2B|A  MHHHHHHSSGVDLGTENL..YFQS.NAMDYFNYQEDGQLWAEQVPLADLANQY.GTPLYV
pdb|4XG1|A  ...........................MDHFNYQNDGRLFVEGLPVEQVVKKT.GTPAYI

                   α2                        β2     η1     α3        β3    
pdb|2YXX|A  20        30                  40        50        60        70
pdb|2YXX|A  YFEETLRKRSRLVKEVFEGV......NLLPTFAVKANNNPVLLKILREEGFGXDVVTKGE
pdb|1KNW|A  YDAQIIRRQIAAL....KQF.......DVVRFAQKACSNIHILRLMREQGVKVDSVSLGE
pdb|5X7M|A  VDEDDFRSRCRDMATAFGGP.......GNVHYASKAFLTKTIARWVDEEGLALDIASINE
pdb|1HKV|A  IDEDDFRSRCRETAAAFGSG.......ANVHYAAKAFLCSEVARWISEEGLCLDVCTGGE
pdb|6N2F|A  YSKPQITRNLEAYKEALEGV......RSVIGYAIKANNNLKILEHLRSLGCGAVLVSGNE
pdb|1TWI|A  MSEEQIKINYNRYIEAFKRWEEETGKEFIVAYAYKANANLAITRLLAKLGCGADVVSGGE
pdb|2QGH|A  YDFDKIKQAFLNYKEAFKGR......KSLICYALKANSNLSILSLLAHLESGADCVSIGE
pdb|2P3E|A  YSSNFIKERFEAYRKAFPD.......ALICYAVKANFNPHLVKLLGELGAGADIVSGGE
pdb|3VAB|A  YSRATIERHFRVFHDAFADM......DTLVTYALKANSNQAVLTALAKLGAGADTVSQGE
pdb|3N2B|A  YSRATLERHWHAFDKSVGDY......PHLICYAVKANSNLGVLNTLARLGSGFDIVSVGE
pdb|4XG1|A  YSRATIERHWQAFDSAAGKH......PHLICYAVKANSNLAVLNLMARMGSGFDIVSVGE
                                            *

            α4      η2     β4        α5          β5      α6
pdb|2YXX|A          80            90        100       110       120
pdb|2YXX|A  LLAAKLAGVPSHT....VVWNGNGKSRDQXEHFLREDVRIVNVDSFEEXEIWRELNPE..
pdb|1KNW|A  IERALAAGYNPQTHPDDIVFTADVIDQATLERVSELQI.PVNAGSVDMLDQLGQ....VS
pdb|5X7M|A  LGIALAAGFPASR....ITAHGNNKGVEFLRALVQNGVGHVVLDSAQELELLDYVAAGEG
pdb|1HKV|A  LAVALHASFPPER....ITLHGNNKSVSELTAAVKAGVGHIVVDSMTEIERLDAIAGEAG
pdb|6N2F|A  LRLALLAGFDPTK....CIFNGNGKSLEDLVLAAQEGV.FVNVDSEFDLNNIVEASRISG
pdb|1TWI|A  LYIAKLSNVPSKK....IVFNGNCKTKEEIIMGIEANIRAFNVDSISELILINETAKELG
pdb|2QGH|A  IQRALKAGIKPYR....IVFSGVGKSAFEIEQALKLNILFLNVESFMELKTIETIAQSLG
pdb|2P3E|A  LYLAKKAGIPPER....IVYAGVGKTEKELTDAVDSEIILMFNVESRQELDVLNEIAGKLG
pdb|3VAB|A  IRRALAAGIPANR....IVFSGVGKTPREMDFALEAGIYCFNVESEPELEILSARAVAAG
pdb|3N2B|A  LERVLAAGGDPSK....VVFSGVGKTEAEMKRALQLKIKCFNVESEPELQRLNKVAGELG
pdb|4XG1|A  LMRVIQAGGDPKK....IVFSGVGKTEIEISAALQANIMCFNVESISELYRINSVAKALN

                 β6              α7          β7 η3         α8        β8
pdb|2YXX|A    130       140       150       160       170
pdb|2YXX|A  .GVEYFIRVNPEVDAKTHPHISTGLKKHKFGIPLEDLDS.....FXERFRSXNIRGLHVH
pdb|1KNW|A  PGHRVWLRVNPGFGHGHSQKTNTGGENSKHGIWYTDLPA...ALDVIQRHHLQLVGIHMH
pdb|5X7M|A  KIQDVLIRVKPGIEAHTHEFIATSHEDQKFGFSLASGSAFEAAKAANNAENLNLVGLHCH
pdb|1HKV|A  IVQDVLVRLTVGVEAHTHEFISTAHEDQKFGLSVASGAAMAAVRRVFATDHLRLVGLHSH
pdb|6N2F|A  KQVNVLLRINPDVDPQVHPYVATGNKNSKFGIRNEKLQWF.LDEVKAHPKELKLVGAHCH
pdb|1TWI|A  ETANVAFRINPNVNPKTHPKISTGLKKNKFGLDVESGIAMKAIKMALEMEYVNVVGVHCH
pdb|2QGH|A  IKARISIRINPNIDAKTHPYISTGLKENKFGVGEKEALE..MFLWAKKSAFLEPVSVHFH
pdb|2P3E|A  KKARIAIRVNPDVDPKTHPYIATGMQKSKFGVDIREAQK..EYEYASKLENLEIVGIHCH
pdb|3VAB|A  KVAPVSLRINPDVDAKTHAKISTGKSENKFGIPRDKARA..AYARAASLPGLNVVGIDMH
pdb|3N2B|A  VKAPISLRINPDVDAKTHPYISTGLRDNKFGITFDRAAQ..VYRLAHSLPNLDVHGIDCH
pdb|4XG1|A  VKAPISIRINPNIDAGTHPYISTGLKENKFGIEIEQALD..VYKIASDLEFLEIKGVDCH
                                                                       *
```

```
                        α9                                  β9
pdb|2YXX|A       180       190       200                    210        220
pdb|2YXX|A  IGSQITRVEPFVEAFSKVVRASE..........RYGFEEINIGGGWGINYSGEELDL...
pdb|1KNW|A  IGSGVDYAH.LEQVCG...AMVRQVIE.....FGQDLQAISAGGGLSVPYQQGEEAVDTE
pdb|5X7M|A  VGSQVFDAEGFKLAAERVLGLYSQIHSELGV....ALPELDLGGGYGIAYTAAEEPLN.V
pdb|1HKV|A  IGSQIFDVDGFELAAHRVIGLLRDVVGEFGPEKTAQIATVDLGGGLGISYLPSDDPPP.I
pdb|6N2F|A  LGSTITKVDIFRDAAVLMIEYIDEIRR.....QGFEVSYLNIGGGLGIDYYHAGAVLPTP
pdb|1TWI|A  IGSQLTDISPFIEETRKVMDFVVELKE.....EGIEIEDVNLGGGLGIPYYKDKQIP.TQ
pdb|2QGH|A  IGSQLLDLEPIIEASQKVAKIAKSLIA.....LGIDLRFFDVGGGIGVSYENEETIK..L
pdb|2P3E|A  IGSQILDISPYREAVEKVVSLYESLTQ.....KGFDIKYLDIGGGLGIKYKPEDKEP.AP
pdb|3VAB|A  IGSQIIDLEPFDNAFALMAELVKELQA.....DGHNIRHVDVGGGLGIPYRTPNTPPPP
pdb|3N2B|A  IGSQLTALAPFIDATDRLLALIDSLKA.....EGIHIRHLDVGGGLGVVYRDELPPQ..P
pdb|4XG1|A  IGSQLTEIAPFIEALDKLLILIDLLAE.....KGITISHLDLGGGLGVPYDDETPPE..P

            α10       η4                 β10      α11  η5        β11
pdb|2YXX|A   230                240        250        260          270
pdb|2YXX|A  SSYREKVV....PDLKR......FKRVIVEIGRYIVAPSGYLLLRVVLVK.....RRHNK
pdb|1KNW|A  HYYG..LWNAAREQIARHL..GHPVKLEIEPGRFLVAQSGVLITQVRSVK.....QMGSR
pdb|5X7M|A  AEVASDLLTAV.GKMAAEL.GIDAPTVLVEPGRAIAGPSTVTIYEVGTTKDVHVDDDKTR
pdb|1HKV|A  AELAAKLGTIV.SDESTAV.GLPTPKLVVEPGRAIAGPGTITLYEVGTVKDVDVSATAHR
pdb|6N2F|A  MDLINT.V....RELVLSRD....LNLIIEPGRSLIANTCCFVNHVTGVK.....TNGTK
pdb|1TWI|A  KDLADAII....NTMLKYKDKVEMPNLILEPGRSLVATAGYLLGKVHHIK.....ETPVT
pdb|2QGH|A  YDYAQGIL....NAL.QGLD....LTIICEPGRSIVAESGELITQVLYEK.....KAQNK
pdb|2P3E|A  QDLAD.LL....KDLLENVK....AKIILEPGRSIMGNAGILITQVQFLK.....DKGSK
pdb|3VAB|A  VAYAQ.IV....AKHIKPLG....LKTVFEPGRLIVGNAGLLVTEVIFVK.....EGDAK
pdb|3N2B|A  SEYAKALL....DRLERHRD....LELIFEPGRAIAANAGVLVTKVEFLK.....HTEHK
pdb|4XG1|A  AEYMTAII....NRMA.GRS....LKLIFEPGRAIMANAGVLVTKVEFLK.....LNDYK
                                     *  *

            β12         α12      β13            β14
pdb|2YXX|A          280       290       300           310        320
pdb|2YXX|A  AFVVVDGGXNVLIRPALYSAYHRIFVLGKQGK......EXRADVVGPLCESGDVIAY...
pdb|1KNW|A  HFVLVDAGFNDLMRPAMYGSYHHISALAADGRSLEHAPTVETVVAGPLCESGDVFTQQEG
pdb|5X7M|A  RYIAVDGGMSDNIRPALYGSEYDARVVSRFAE....GDPVSTRIVGSHCESGDILIN...
pdb|1HKV|A  RYVSVDGGMSDNIRTALYGAQYDVRLVSRVSD....APPVPARLVGKHCESGDIIVR...
pdb|6N2F|A  NFIVIDGSMAELIRPSLYDAYQHIELVSPTPP...EAEVTKFDVVGPVCESADFLGK...
pdb|1TWI|A  KWVMIDAGMNDMMRPAMYEAYHHIINCKVKNE......KEVVSIAGGLCESSDVFGR...
pdb|2QGH|A  RFVIVDAGMNDFLRPSLYHAKHAIRVITPSKG....REISPCDVVGPVCESSDTFLK...
pdb|2P3E|A  HFIIVDAGMNDLIRPSIYNAYHHIIPVETKER.....KKVVADIVGPICETGDFLAL...
pdb|3VAB|A  NFVIVDAAMNDLIRPTLYDAFHDIRPVIMPND...NAPRIRADFVGPVCETGDYLGL...
pdb|3N2B|A  NFAIIDAAMNDLIRPALYQAWQDIIPLRPRQG.....EAQTYDLVGPVCETSDFLGK...
pdb|4XG1|A  NFAIVDAAMNDLIRPALYSAWQNIIPLNTDYQDGQDRPVRSYDIVGPICETGDFLGK...
                          *                                *

            β15               β16        η6          β17        β18
pdb|2YXX|A  ....            330       340       350       360       370
pdb|2YXX|A  ....DRELPE.VEPGDIIAVENAGAYGYTXSNNYNSTTRPAEVLVRENGRISLIRRRETE
pdb|1KNW|A  GNVETRALPE.VKAGDYLVLHDTGAYGASMSSNYNSRPLLPEVLFDN.GQARLIRRRQTI
pdb|5X7M|A  ....DEIYPSDITSGDFLALAATGAYCYAMSSRYNAFTRPAVVSVRA.GSSRLMLRRETL
pdb|1HKV|A  ....DTWVPDDIRPGDLVAVAATGAYCYSLSSRYNMVGRPAVVAVHA.GNARLVLRRETV
pdb|6N2F|A  ....DRELPT.PPQGAGLVVHDAGAYCMSMASTYNLKMRPPEYWVEEDGSITKIRHAETF
pdb|1TWI|A  ....DRELDK.VEVGDVLAIFDVGAYGISMANNYNARGRPRMVLTSK.KGVFLIRERETY
pdb|2QGH|A  ....DAHLPE.LEPGDKIAIEKVGAYGSSMASQYNSRPKLLELALED.HKIRVIRKREAL
pdb|2P3E|A  ....DREIEE.VQRGEYLAVLSAGAYGFAMSSHYNMRPRAAEVLVEN.GSVKLIRKRENY
pdb|3VAB|A  ....DREVAK.PAPGDLIAICTTGAYGAVLSSTYNSRLLIPEVLGDG.ERYHVVRPRRTY
pdb|3N2B|A  ....DRDLVL..QEGDLLAVRSSGAYGFTMSSNYNTRPRVAEVMVDG.NKTYLVRQREEL
pdb|4XG1|A  ....ERQLAL..AEGDYLVIRSTGAYGSTMSSNYNSRCRAAEILVDG.EKAFIVREREEL
```

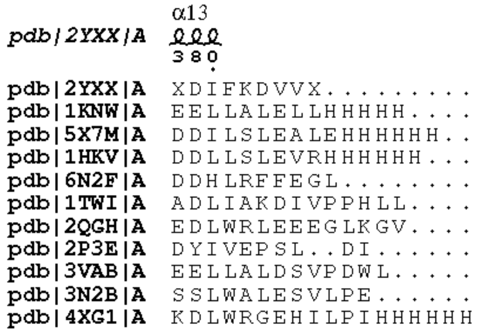

Figure S5: **Sequence alignment of 11 different DAPDCs.** 2YXX: *T. maritima*; 1KNW: *E. coli*; 5X7M: *C. glutamicum*; 1HKV: *M. tuberculosis*; 6N2F: *A. thaliana*; 1TWI*: M. jannaschii*; 2QGH: *H. pylori*; 2P3E: *A. aeolicus*; 3VAB: *B. melitensis*; 3N2B: *V. cholerae*; 4XG1: *P. ingrahamii*. Identical residues are marked in red. Residues marked with * belong to the active site. Other conserved motifs contribute to formation of the (α/β)$_8$-TIM barrel or to PLP binding, like HI/VGS (179–182), the glycine-rich region (212-214), EI/PGRS (246– 249), and GAY (341-343).

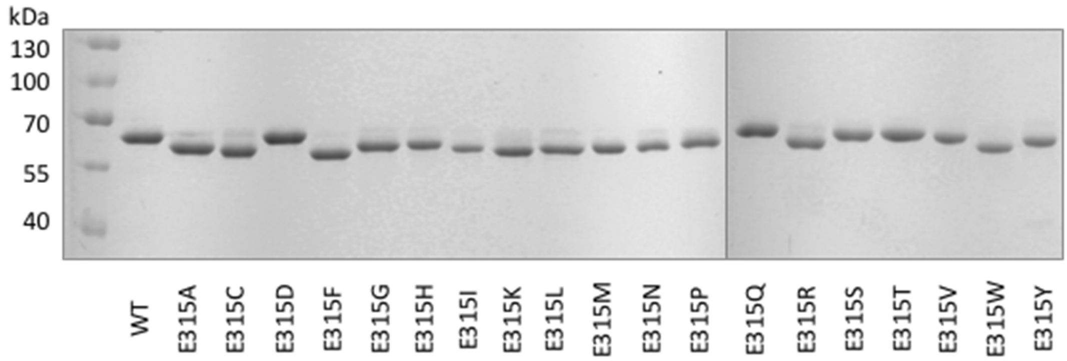

Figure S6: Reductive SDS-PAGE gel of IMAC-purified mutant DAPDCs. Marker: PageRuler prestained protein ladder (Thermo Scientific).

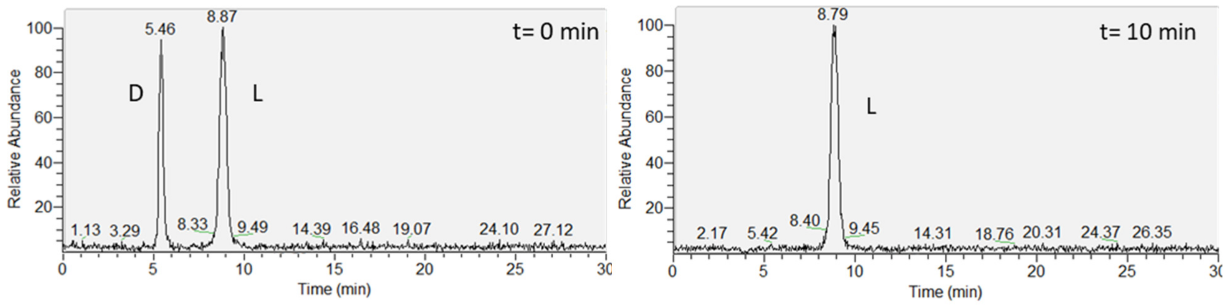

Figure S7. Kinetic resolution of racemic 2-APA by the *Tm*DAPDC E315T mutant. The panels show LC-MS spectra of a reaction mixture at t=0 and t=10 min.

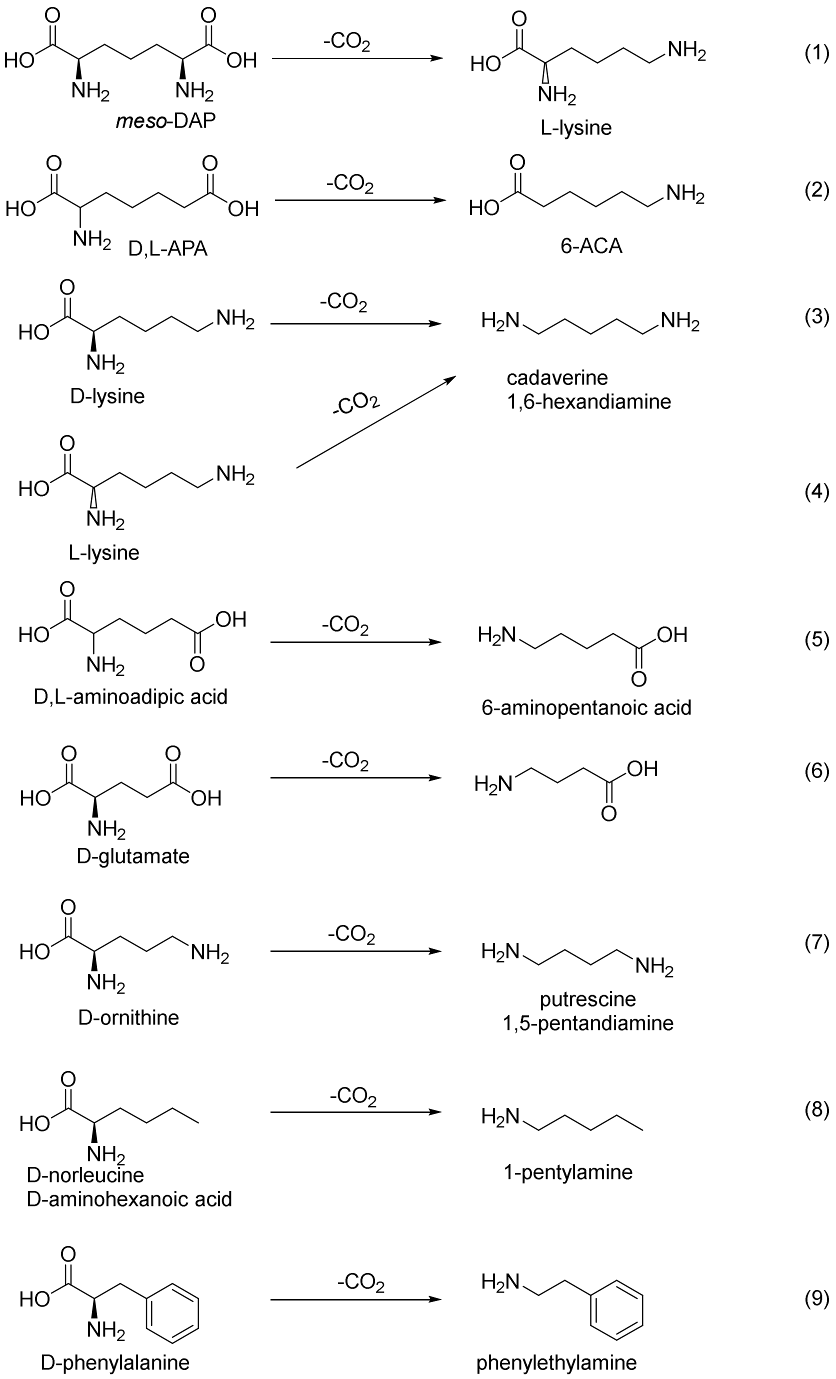

Figure S8: **Substrates tested with the mutant library E315X.** Substrates that were picked have either structural similarity to DAP or 2-APA with variations in side-chain length (aminoadipic acid), functional groups (lysine), hydrophobicity or bulkiness (D-phenylalanine).

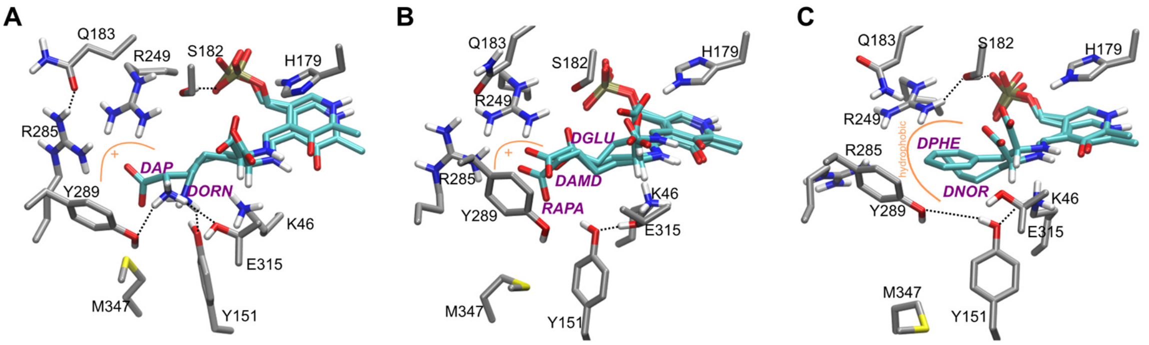

Figure S9: Docked structures of several ligands into the binding site of variant E315T. The ligands shown are the external aldimine intermediates of the compounds presented in Table 2. Abbreviations: DAP, *meso*-DAP; RAPA, D,L-2-APA; DAMD, D,L-α-aminoadipic acid; DGLU, D-glutamate; DORN, D-ornithine; DNOR, D-norleucine; DPHE, D-phenylalanine. **A**: D-ornithine lacks a distal carboxylate that can form salt bridges with R249 and R285, as is the case with *meso*-DAP. **B**: The difference between D,L-2-APA, D,L-α-aminoadipic acid, and D-glutamate is the side chain length, and hence the potential to form salt bridges between the distal carboxylate and R249/R285. The enzyme presents high activity toward the first, barely observable activity toward the second, and no observable activity toward the third compound. **C**: The binding site of DAPDC does not accommodate well the bare phenyl or alkyl chains of D-phenylalanine and D-norleucine, respectively. No detectable activity was found across the E315X variants toward the two hydrophobic compounds, with the exception of the more hydrophobic variants E315P and E315L, where some activity was detected.

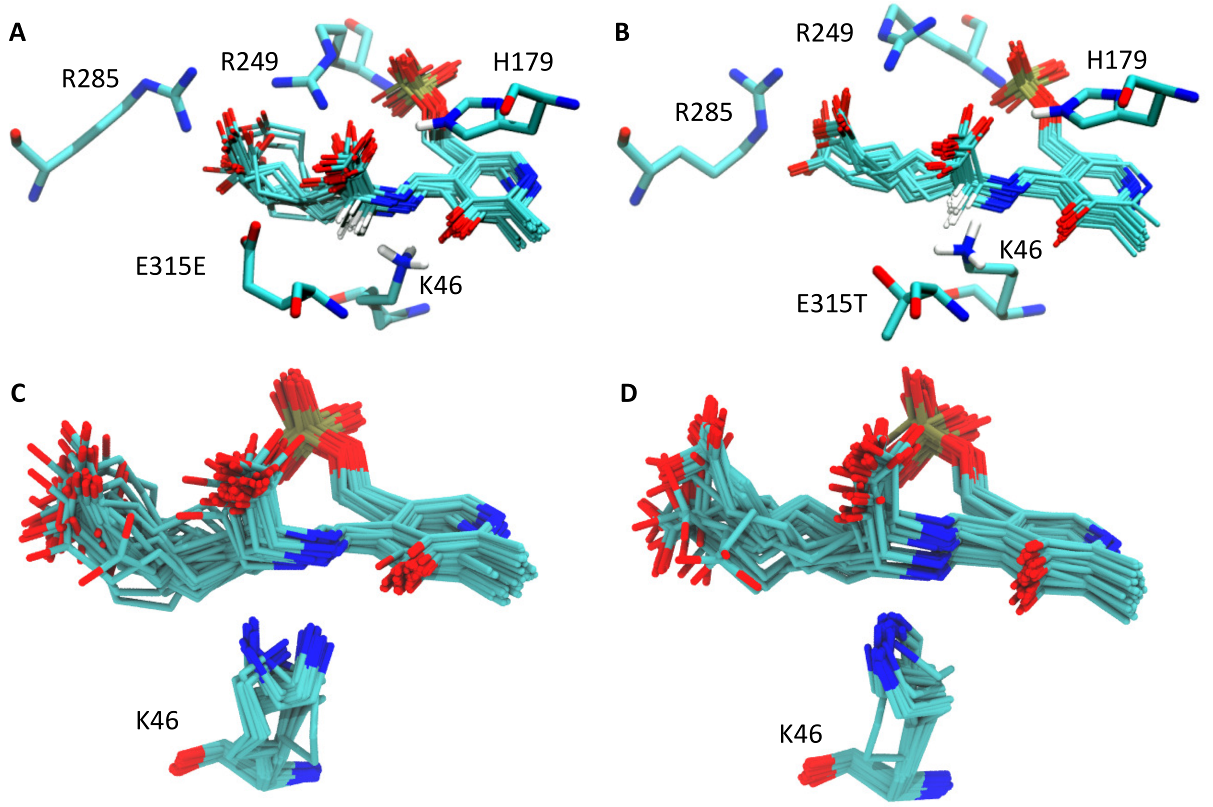

Figure S10: **Docking poses overlap of the (*R*)-APA external aldimine docked in DAPDC obtained by Rosetta. A** and **C**: (*R*)-2-APA in wild-type DAPDC (E315E). **B** and **D**: (*R*)-2-APA in mutant E315T. In both variants (E315E and E315T), salt bridges are present between the distal carboxylate of (*R*)-2-APA and two active site arginine residues, Arg429 and Arg285. For enzyme variant E315T, Rosetta produced structures where the catalytic lysine Lys46 adopts a conformation that positions its ε-amino group closer to the C_α atom, making the electrophilic substitution more likely to occur than in E315E. In E315T, the ε-amino group of Lys46 forms a hydrogen bond with the hydroxyl group of Thr315.

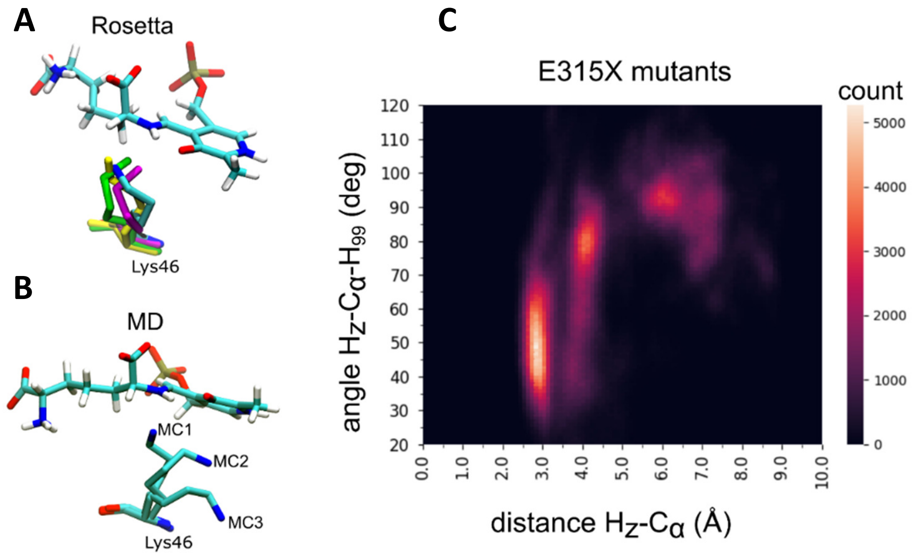

Figure S11: Conformations adopted by the catalytic Lys46 in docking and MD simulations. Binding poses are predominantly determined by the conformation of Lys46 (d_1, θ_1). **A**: Lys46 conformations produced in the docking stage (denoted in yellow, green, magenta, and cyan colors) that evolved during the equilibration stage to become either one of three distinct conformations. **B:** The three observed Lys46 conformations, denoted as MC1-MC3, with distinct distances to the substrate's C_α atom. Conformations MC1-MC3 were adopted during the early equilibration stage and rarely interchanged during the short MD runs. **C:** Heatmap of the population of the Lys46 conformations found in the trajectories. Conformation MC1 produced more reactive binding poses than MC2 and MC3. Mutants with higher percentage of reactive binding poses had a larger population of MC1 conformations.
